# The effects of promoting oral intake using the Kuchi-kara Taberu index, a comprehensive feeding assistant tool, in older pneumonia patients: a cluster randomized controlled trial

**DOI:** 10.1186/s12877-020-1447-x

**Published:** 2020-01-31

**Authors:** Hiroshi Shamoto, Tamami Koyama, Ryo Momosaki, Keisuke Maeda, Hidetaka Wakabayashi

**Affiliations:** 1 Department of Geriatric Internal Medicine, Takano Hospital, 214 Higashi-machi, shimokitaba, Hirono-machi, Futaba-County, Fukushima, 960-0402 Japan; 2 Department of Disaster and Comprehensive Medicine, Fukushima Medical University, 1 Hikarigaoka, Fukushima City, Fukushima, 960-1295 Japan; 3 Chairman, Kuchi-kara Taberu Shiawase-wo Mamoru-kai (KTSM, an incorporated nonprofit organization), 509, 722-1,Ishida, Isehara City, Kanagawa 259-1116 Japan; 4 Department of Rehabilitation Medicine, Teikyo University School of Medicine University Hospital, Mizonokuchi, 5-1-1 Futako, Takatsu-ku, Kawasaki, Kanagawa 213-8507 Japan; 5 Department of Palliative and Supportive Medicine, Graduate School of Medicine, Aichi Medical University, 1-1, Yazakokarimata, Nagakute City, Aichi 480-1195 Japan; 6 Department of Rehabilitation Medicine, Yokohama City University Medical Center, 4-57 Urafune-chou, Minami ward, Yokohama City, Kanagawa 232-0024 Japan

**Keywords:** Deglutition, Deglutition disorder, Eating, Nutrition therapy, Pneumonia, Rehabilitation

## Abstract

**Background:**

The multidisciplinary comprehensive care (MDCC) program promotes the improvement of oral intake for older patients. The Kuchi-kara Taberu (ingesting orally in Japanese, KT) index was developed to objectively assess patient conditions in the MDCC program. This trial examined the effects of the index in promoting oral intake in older patients with pneumonia.

**Methods:**

A cluster randomized controlled trial was conducted in 10 local hospitals targeting older patients with pneumonia (≥65 years). Ten hospitals were allocated randomly to either the intervention or the control group. Both groups (each with five hospitals) received the MDCC program for oral feeding, which consisted of professional assessment, care, and treatment. The KT index was used by the intervention group, focusing on improving low score items. The primary outcome was determined using the Functional Oral Intake Scale (FOIS) at discharge or 1 month after admission.

**Results:**

One hundred and twelve patients (46 women and 66 men) who participated from 10 hospitals, with a median age of 88 years (interquartile range [IQR], 80–91), were examined. The median FOIS level and the number of patients with oral intake (FOIS ≥ level 4) at discharge were 4 (IQR, 4–6) and 89 (79.5%), respectively. The duration of *nil* per os was 2 (IQR, 1–5) days. Clusters were not matched in the presence of Kuchi-kara Taberu Shiawase-wo Mamoru-kai-certified medical staff promoting oral intake in patients with dysphagia in each hospital. The median FOIS levels of 53 patients in the intervention group and 59 patients in the control group were 5 (IQR, 4–6) and 4 (IQR, 4–5), respectively, showing no statistically significant difference (*P =* 0.76). According to a multivariate analysis, the KT index had no positive effect on FOIS levels.

**Conclusions:**

This trial was not able to demonstrate the usefulness of the KT index due to random assignment failure. However, both the intervention and control groups showed a high prevalence of oral intake (FOIS ≥ level 4) at discharge.

**Trial registration:**

UMIN-Clinical Trial Registry, UMIN000025172, December 17, 2016.

## Background

Pneumonia together with aspiration pneumonia is the third leading cause of death in Japan [1]. Approximately 95% of pneumonia deaths are observed among older people aged 65 years and over, and the majority of these patients experience the complications of deglutition disorder [2]. A previous study revealed that aspiration pneumonia may result in further deterioration of swallowing function and malnutrition due to *nil* per os after hospitalization [3]. Other studies have also indicated that deglutition disorder is significantly associated with pneumonia [4, 5].

Meanwhile, an early intervention program for oral feeding, which includes not only dysphagia rehabilitation but also early physical therapy [6], daily oral care [7], and food modification [8], has been reported to reduce mortality rates in older patients with severe pneumonia and the incidence of pneumonia in hospitalized older patients. Older patients with pneumonia may have a limited swallowing functional reserve and can easily develop swallowing dysfunction [8]; therefore, comprehensive support is required for the early resumption of oral intake. The multidisciplinary comprehensive care (MDCC) is an intervention program based on aging assessment and aimed at multifaceted assessment and treatment for frail older patients. This program has been proven to be beneficial in the clinical setting [9].

We previously reported that a MDCC program for oral feeding, which we had developed for older patients with pneumonia, may promote early recommencement of oral intake during hospitalization and shortened the length of hospital stay [10]. The MDCC program promotes the improvement of oral intake, based on careful identification and assessment of the patient’s problems related to physical function and dietary intake, and resolves medical, physical, social, and cognitive problems [10]. The program often requires a leading person (a specialized nurse) for adequate assessment of patient conditions and problems to provide MDCC with the cooperation of other healthcare staff [10]. The Kuchi-kara Taberu (ingesting orally in Japanese, KT) index was developed to be used in the MDCC program for every medical or care staff without a leading person to easily and objectively assess patient conditions, including swallowing function, activity, nutrition, health condition, and cognition [11, 12]. The reliability and validity of the KT index have already been confirmed in a past study [13].

Although a previous case report and case control study demonstrated the usefulness of the KT index for older patients with dysphagia [14, 15], interventional studies investigating the effectiveness of the KT index in older patients with pneumonia have not been conducted yet. Thus, the current intervention study aimed to verify whether the MDCC program using the KT index promotes oral intake in older patients with pneumonia.

## Methods

### Study design

This was a single-blinded cluster randomized controlled trial (RCT). Study enrollment and data collection started in March 2017 and ended in February 2018. All participants were recruited by research coworkers through an incorporated nonprofit organization, Kuchi-kara Taberu Shiawase-wo Mamoru-kai (KTSM, the Association for the Preservation of Pleasure Experienced through Oral Intake of Food and Drink), which was established in June 2013.

The trial was approved by the Ethics Committee of Minamisoma Municipal General Hospital on October 13, 2016 (Ethics Committee Authorization Number 28–11) and conducted in accordance with the ethical standards of the 1964 Declaration of Helsinki and its later amendments. All participants provided written informed consent. The study was registered in the University Hospital Medical Information Network (UMIN) Clinical Trial Registry (UMIN000025172). This clinical trial followed the Consolidated Standards of Reporting Trials (CONSORT) statement on randomized trials of non-pharmacological treatment [16].

### Randomization

Ten hospitals were allocated randomly to either the intervention or the control group. Permuted block randomization with block sizes of two and four was performed by a research coordinator blinded to data collection and analysis of the research work. The blocks were assigned randomly using a computer-generated random number table. Group allocation was concealed from all hospitals. The medical staff and participants were not blinded to their group allocation.

### Participants

Patients 65 years and older admitted to the hospitals with pneumonia were enrolled in the study. Patients without ordinary food intake at admission, with mild pneumonia (A-DROP Score 0, described later), who did not agree to participate in the trial, and who had not been referred to an MDCC team were excluded.

The severity of pneumonia was assessed using the Japanese version of the CURB-65 severity score by A-DROP assessment [17]. The A-DROP score composed of 5 factors as follows: the patients’ age (men, ≥70 years; women, ≥75 years), dehydration status (blood urea nitrogen ≥21 mg/dL or presence of dehydration), respiratory status (percutaneous oxygen saturation ≤ 90% or arterial oxygen saturation ≤ 60 Torr), level of consciousness, and systolic blood pressure (≤90 mmHg). One point was assigned to each of the factors. The definition of severity was the following: 0 point, mild; 1 or 2 point, moderate; 3 point, severe; 4 or 5 point, super-severe.

### Interventions

The MDCC program with time-series evaluation using the KT index was conducted in the intervention group. The time-series evaluation sheet of the KT index was recorded during a multidisciplinary meeting, which was held once or several times a week until patient discharge. The KT index comprised 13 items as follows: willingness to eat, overall condition, respiratory condition, oral condition, cognitive function while eating, oral preparatory and propulsive phases, severity of pharyngeal dysphagia, position and endurance while eating, eating behavior, daily living activities, food intake level, food modification, and nutrition. Each item was rated from 1 (worst) to 5 (best) points and recorded in a radar chart, which visualized the weak items and the effects of intervention through the time-series charts [11, 12, 14, 15]. The intervention group provided an outline of multidisciplinary opinions and intervention plans according to the results of the KT index at every conference. They also monitored the intervention effect using the KT index radar chart and discussed whether the interventions were appropriate for the patient and created new plans at every conference, if needed.

Both the intervention and control groups received pharmacological therapy or oxygen therapy to improve overall condition and the MDCC program. The MDCC program consisted of professional assessment, care, and treatment. The treatment included dysphagia rehabilitation, maintaining posture and strengthening endurance while eating, improvement of activities of daily living, and improvement of nutritional status paying attention to dietary intake level and food texture. The detailed protocol of the MDCC program was described in our previous report [10]. In the intervention group, the MDCC program was implemented based on objective evaluation using the KT index, focusing on improving low score items and also maintaining and strengthening high score items. The control group received the MDCC program without the KT index evaluation.

### Primary outcomes

Our previous study showed that the median (interquartile range [IQR]) length of hospital stay of older adults with severe pneumonia was 16 (IQR, 11–25) days. Therefore, the Functional Oral Intake Scale (FOIS) [18] was applied at discharge or 1 month after admission to assess swallowing function. The FOIS level ranged from Level 1 (nothing by mouth) to Level 7 (total oral diet with no restrictions).

### Secondary outcomes

Activities of daily living were assessed using the Barthel Index (BI), consisting of feeding, bathing, grooming, dressing, bowel control, bladder control, toileting, chair transfer, ambulation, and stair climbing [19] at discharge. Duration of hospitalization, duration of *nil* per os after admission, resumption of oral intake at discharge (FOIS level ≥ 4), nutritional intake (kcal) at discharge or 1 month after admission, and period of antibiotic administration were also recorded.

### Other parameters

Medical history of cerebrovascular disease, dementia, pneumonia, neurodegenerative diseases, and head and neck cancer was reviewed because any of these diseases can cause premorbid deglutition disorder and be confounding factors potentially affecting FOIS at discharge or 1 month after admission. Data on premorbid BI, nutritional intake (kcal) during the first 24 h after admission, nutritional status at admission (assessed using the Mini Nutritional Assessment Short Form [MNA-SF] including six questions to evaluate the nutritional status and provide intervention as needed) [20], age, sex, home discharge rate, and mortality were collected.

The KTSM produced a specific practical certification system in September 2014 to certify medical or care staff trained for providing feeding support to patients with dysphagia. There were 54 KTSM practical skill-certified individuals in several institutions throughout Japan as of March 2018. These KTSM-certified individuals first went through a document review. The document review required an assessment using the KT index, the planning and implementation of an intervention plan based on the assessment, and the evaluation of the intervention effect using the KT index. Additionally, the evaluation and intervention processes were reviewed, and the appropriateness of the information provided to the team medical members and the patient’s family was examined. Next, they were asked to hold a workshop and to guide the participants. If the content of the instruction was insufficient, the instructor was obliged to participate in the subsequent workshops. Finally, they achieved a certain score (80 points out of 100) in the practical test and oral examination. Even after qualification, obtaining an evaluation score above a certain level at the annual training program and at the renewal practice test and annual qualification renewal seminar every 3 years was necessary. They were mainly involved in the assessment of the patient’s general condition and physical function, dysphagia rehabilitation, and nutritional treatment of the MDCC program. The number and location of KTSM-certified individuals who participated in this RCT and their contribution to the MDCC program were recorded.

### Sample size calculation

A previous study described that the mean FOIS levels at discharge in older people with aspiration pneumonia were 3.97 ± 1.88 in the tentative *nil* per os group and 5.29 ± 1.01 in the early oral intake group [3]. We hypothesized that the usefulness of the KT index would be confirmed if the difference in FOIS levels at discharge between the intervention and control groups was 1.32 or more. The sample size was calculated as 34 per group to obtain a power of 90% with a significance level of 5%. Thus, 34 experimental participants and 34 control participants would be required to be able to reject the null hypothesis. We assumed that each cluster had 10 patients, and a sample size expansion factor was calculated as 1 + (10–1) × (intra-cluster correlation coefficient was assumed to be 0.05 [21] = 1.45). Therefore, the necessary sample size in this RCT was calculated as 68 × 1.45 = 99 patients in 10 hospitals.

### Statistical analysis

The parametric and nonparametric data were presented as means ± standard deviation and median (IQR), respectively. Differences between the intervention group and the control group were analyzed using the Mann-Whitney U test accordingly. Categorical data were expressed as number and percentages, with differences analyzed using the Fisher’s exact test or the chi-squared test.

Considering intra-cluster correlation, the effects of the KT index were examined based on the generalized estimating equation model for the FOIS levels and secondary outcomes, adjusted for facility cluster, age, sex, MNA-SF at admission, pre-morbid BI, and history of diseases. The analyses were performed using the Statistical Package for the Social Sciences version 25.0 software (International Business Machines Corporation, Tokyo, Japan), and *P* values < 0.05 were considered significant.

## Results

**Fig. 1:**
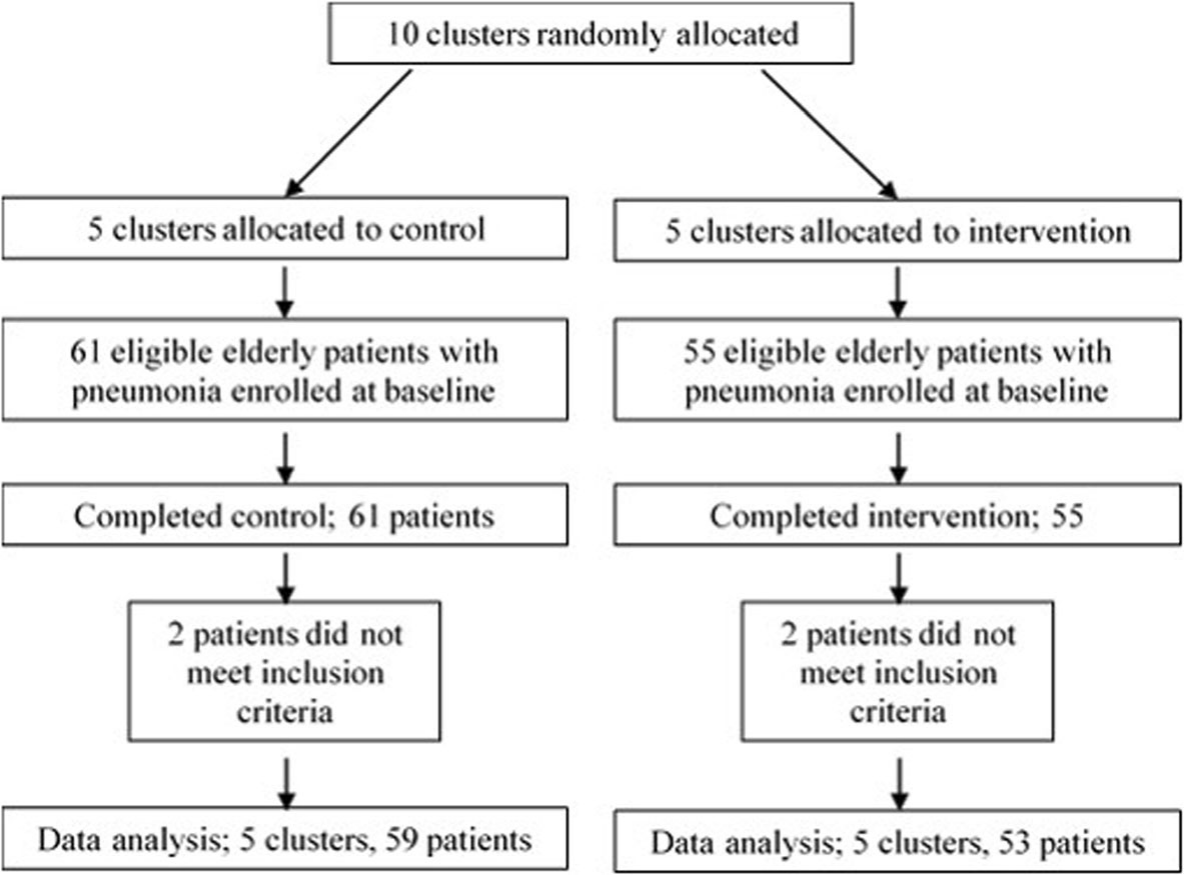
Flow diagram of the participants

Figure 1 shows the flow of clusters and patients in the current trial. Although 116 patients were enrolled and divided into either the intervention or the control group, two patients were excluded from both groups before the analysis due to mild pneumonia.

The characteristics of the 112 patients (66 men and 46 women; median age, 88.0 [IQR, 80.3–91.0] years) and the participating institutions (the number of hospital beds and annual number of pneumonia hospitalized patients) are summarized in Table 1.

**Table 1:** Patient and cluster characteristics (Pre-intervention)

Variables	Total	Intervention	Control	*P* value
	*n* = 112	*n* = 53	*n* = 59	
Age (y), median (IQR)	88 [80–91]	89 [83–91]	87 [80–91]	0.46
Gender (Male)	66 (58.9%)	27 (50.1%)	39 (66.1%)	0.08
Pre-morbid residence Home	50 (44.6%)	20 (37.7%)	30 (50.8%)	0.21
Hospital	1 (0.9%)	0 (0%)	1 (1.7%)	
Institution	61 (54.5%)	33 (62.3%)	28 (47.5%)	
A-DROP Very severe	2 (1.8%)	2 (3.8%)	0 (0%)	0.27
Severe	31 (27.7%)	13 (24.5%)	18 (30.5%)	
Moderate	79 (70.5%)	38 (71.7%)	41 (69.5%)	
Stroke	49 (43.8%)	28 (52.8%)	21 (35.6%)	0.05
Dementia	69 (61.6%)	39 (73.6%)	30 (50.9%)	0.01
Pneumonia	65 (58.0%)	28 (52.8%)	37 (62.7%)	0.19
Neurodegenerative	10 (8.9%)	1 (1.9%)	9 (15.3%)	0.01
Head and neck tumor	1 (0.9%)	0 (0%)	1 (1.7%)	0.53
MNA-SF, median (IQR)	6 [5–8]	6 [5–9]	6 [5–8]	0.9
Barthel Index, median (IQR)	20 [0–60]	15 [5–50]	30 [0–60]	0.45
Nutrition intake (kcal) during first 24 h after admission, median (IQR)	260 [175–833]	544 [161–1095]	258 [210–420]	0.02
Hospital beds, median (IQR)		150 [61.5–378.5]	144 [71.5–376.0]	0.92
Annual pneumonia patients, median (IQR)		100 [46.5–200.0]	140 [87.0–311.5]	0.47
KTSM certified people (%)		1 (20%)	5 (100%)	0.05

*Abbreviations*: *IQR* interquartile range,

A-DROP the severity of pneumonia determined using the Japanese version of the CURB-65 severity score, MNA-SF mini nutritional assessment-short form,

Both groups were well matched except for past medical history (dementia and neurodegenerative disease), total nutritional intake during the first 24 h after admission, and the number of KTSM practical skill-certified individuals. In the intervention group, 73.6% of the patients were diagnosed with dementia, whereas 50.1% were diagnosed with dementia in the control group. There were differences in total nutritional intake during the first 24 h after admission and the number of KTSM-certified individuals between the two groups. The total nutritional intakes were 544 (IQR, 161–1095) kcal in the intervention group and 258 (IQR, 210–420) kcal in the control group. In the control group, each of the five hospitals had one or more KTSM-certified individuals who played a leading role in the MDCC program, such as dysphagia rehabilitation and nutritional treatment. On the contrary, in the intervention group, only one KTSM-certified individual was available in one hospital.

Table 2 shows the outcomes of the 112 participants in both groups. The median FOIS level at discharge or 1 month after admission, duration of *nil* per os, and oral intake (FOIS ≥ level 4) at discharge were 4 (IQR, 4–6), 2 (IQR, 1–5) days, and 89 (79.5%) patients, respectively.

**Table 2:** The univariate analysis of outcomes after intervention in both intervention and control groups

Variables	Total	Intervention	Control	*P* value
	*n* = 112	*n* = 53	*n* = 59	
FOIS level at dischrge/1 month after admission, median (IQR)	4 [4–6]	5 [4–6]	4 [4–5]	0.763
Barthel Indexat dischrge/1 month after admission, median (IQR)	15 [0–50]	15 [0–45]	15 [0–55]	0.754
Duration of *Nil* per Os (days)after admission, median (IQR)	2 [1–5]	1 [0–5]	3 [1–6]	0.002
Oral intake (FOIS level 4 or more) at dischrge/1 month after admission, median (IQR)	89 (79.5%)	42 (79.2%)	47 (79.7%)	0.57
Nutrition intake (kcal)at dischrge/1 month after admission, median (IQR)	1200 [803–1400]	1100 [740–1400]	1200 [900–1400]	0.431
Duration of antibiotics (days), median (IQR)	9 [7–13]	8 [7–12]	9 [7–14]	0.262
Post-discharge residence Home	35 (31.3%)	15 (28.3%)	20 (33.9%)	0.321
Hospital	26 (23.2%)	10 (18.9%)	16 (27.1%)	
Institution	51 (45.5%)	28 (52.8%)	23 (39.0%)	
Number of deaths	11 (9.8%)	6 (11.3%)	5 (8.5%)	0.424

*Abbreviations*: *FOIS* Functional Oral Intake Scale, *IQR* interquartile range

There was no significant difference associated with FOIS level at discharge or 1 month after admission in either univariate analysis (median FOIS level: intervention group, 5 [IQR, 4–6]; control group, 4 [IQR, 4–5]; *P =* 0.763) (Table 2) or multivariate analysis based on the generalized estimating equation model (beta = 0.016; 95% confidence interval [CI], − 0.465–0.498; *P =* 0.946) (Table 3). Meanwhile, the multivariate analysis indicated that MNA-SF score at admission was significantly associated with the FOIS at discharge or 1 month after admission (beta = 0.169; 95% CI, 0.03–0.309; *P =* 0.017).

**Table 3:** The effects of the KT-index in the generalized estimating equation model for FOIS level

FOIS level	beta	Standard error	95% Confidence interval of beta	*P* value
Constant	1.343	1.541	(−1.677 to 4.362)	0.383
Intervention	0.016	0.246	(−0.465 to 0.498)	0.946
Age	0.015	0.013	(−0.01 to 0.04)	0.239
Gender	0.363	0.433	(− 0.487 to 1.212)	0.403
Past medical history	−0.375	0.392	(−1.144 to 0.394)	0.339
MNA-SF	0.169	0.071	(0.03 to 0.309)	0.017
Pre-morbid Barthel Index	0.011	0.009	(−0.007 to 0.028)	0.227

*Abbreviations*: *FOIS* functional oral intake scale, *MNA-SF* mini nutritional assessment-short form

In the secondary outcomes, significant differences were observed in the duration of *nil* per os after admission (*P =* 0.002), but not in other outcomes (BI at discharge, duration of hospitalization, resumption of oral intake at discharge, nutritional intake [kcal] at discharge or 1 month after admission, and period of antibiotic administration), with the univariate analysis (Table 2). However, regarding the multivariate analysis, based on the generalized estimating equation model, there were no significant differences in the duration of *nil* per os after admission (beta = − 2.786; 95% CI, − 0.6963–1.390; *P =* 0.191), as was the case in all other outcomes (Table 3).

## Discussion

This study is a cluster RCT examining whether the MDCC program using the KT index for older patients with pneumonia could promote resumption of oral intake. The results did not show positive effects of the KT index on FOIS levels, probably due to random assignment failure.

A relatively higher level of feeding support care might have been provided in all participated institutions. In both the intervention and control groups, the total oral intake ratio at discharge or 1 month after admission (FOIS ≥level 4) was 79.5%, higher than that in a previous study [22]. The rate of total oral intake at 1 month after admission in patients aged ≥65 years with pneumonia was 59.0% in a previous study according to the data from the Japanese Diagnosis Procedure Combination inpatient database [22]. Furthermore, the period between admission and resumption of oral intake in the control group was 3 (IQR, 1–6) days. Duration of days until initial oral intake was 4.8 ± 4.9 days or 4 days (IQR, 3–6) in the non-early oral intake group of the previous cohort studies in Japan [3, 23]. There have been several reports concerning an impact on the clinical outcomes of early oral intake after admission. Early oral intake following gastrointestinal surgery has been reported to reduce the number of hospital-acquired pneumonia cases [24]. In our previous study, oral intake within 2 days after admission shortened the length of hospital stay and increased the complete oral intake rate at discharge [23]. Additionally, Maeda and colleagues reported that early oral intake shortened the treatment period, improved swallowing function, and reduced mortality [4].

The current study design might be inadequate, and it was not possible to consider the distribution and number of KTSM-certified individuals, which would have the largest difference between clusters. In the present trial, all institutions allocated for the control group had medical staff considered to be KTSM-certified individuals feeding support for patients with dysphagia. KTSM-certified individuals achieve a comprehensive perspective through multiple practical trainings and practical trainings. It can be suggested that the MDCC program conducted with a KTSM-certified individual might have already been established and provide effective feeding support for patients with dysphagia similar to the MDCC program conducted using the KT index. Therefore, even without the KT index, the oral intake resumption rate might have already been high before starting the current trial, and there might have been no difference in the improvement of oral intake between the two groups. Using stratified randomization with equal distribution of the assignment of KTSM-certified individuals to observe the true effect of the KT index might have been required in the current trial.

The previous studies have shown that using the KT index can assess patients from a comprehensive perspective and increase their subsequent care and treatment effectiveness in various settings such as the acute phase, convalescent phase, and nursing home [13–15]. On the contrary, if intensive treatment for severe pneumonia, severe complications of pneumonia, or severely impaired consciousness is prioritized, the KT index may not be applicable. We hypothesized that the KT index may not be beneficial in healthcare facilities where KTSM-certified individuals lead the team medical care. However, since these certified individuals are not assigned to all healthcare facilities, we believe that it will be necessary to confirm the usefulness of the KT index in several clinical settings and diseases in the future.

The prevalence of dementia was significantly higher in the intervention group than that in the control group. Therefore, in the intervention group, dementia might have negatively influenced the effect of the intervention. A previous study has suggested that dementia can interfere with dysphagia rehabilitation [25]. Meanwhile, our previous study has demonstrated that the severity of dementia does not affect the effect of dysphagia rehabilitation [26]. We hypothesized that the presence of dementia was an unresolved issue in the healthcare setting, and further double-blind randomized controlled studies should be conducted with careful consideration of the presence of dementia.

In the present study, nutritional condition at admission was independently associated with the FOIS level at discharge or 1 month after admission. This is consistent with the results of the previous trials, indicating that the better nutritional status was associated with better outcomes for dysphagia rehabilitation or better swallowing function both in the clinical and nursing care settings [27, 28].

The present study has some limitations. The medical staff at the participating institutions were not blinded to group allocation. Some medical staff in the control group may have enhanced the participants’ enthusiasm about working on rehabilitation treatment. Thus, some participants in the control group may have better outcomes at discharge than desired. The quality of the MDCC program among the participating institutions was not assessed in the current trial. However, we hypothesized that the adequate MDCC program for oral feeding was provided in all the participating institutions.

## Conclusions

The usefulness of the KT index was not demonstrated in the current RCT. However, both groups showed a high prevalence of oral intake (FOIS ≥ level 4) at discharge.

## Data Availability

The datasets used and/or analyzed during the current study are available from the corresponding author on a reasonable request.

## Abbreviations

BI: Barthel index
FOIS: Functional oral intake scale
KT index: Kuchi-kara Taberu index
KTSM: Kuchi-kara Taberu Shiawase-wo Mamoru-kai in Japanese (The Association for the Preservation of Pleasure Experienced through Oral Intake of Food and Drink)
MDCC: Multidisciplinary comprehensive care
MNA-SF: Mini nutritional assessment short form
